# Supplementary material for: Giant excitonic absorption and emission in two-dimensional group-III nitrides
Source: Sci Rep. 2020 Jul 1;10:10719. doi: 10.1038/s41598-020-67667-2 (PMC7329854; doi:10.1038/s41598-020-67667-2)
Supplement: Supplementary file 1 — Supplementary information [file 41598_2020_67667_MOESM1_ESM.pdf]

Supplemetal Material for ”Giant excitonic absorption and emission in two-dimensional group-III nitrides”

Maria Stella Prete[1], Davide Grassano[1], Olivia Pulci[1,*], Ihor Kupchak[2], Valerio Olevano[1,3], and Friedhelm Bechstedt[4]

[1]Dipartimento di Fisica, Università di Roma Tor Vergata, and INFN, Via della Ricerca Scientifica 1, I-00133 Rome, Italy
[2]V.E. Lashkaryov Institute of Semiconductor Physics, National Academy of Sciences of Ukraine, Kyiv, Ukraine
[3]CNRS, LPMMC, 38042 Grenoble, France
[4]IFTO, Friedrich Schiller Universität, Max-Wien Platz 1, D-07743 Jena, Germany
[*]olivia.pulci@roma2.infn.it

ABSTRACT

Absorption and emission of pristine-like semiconducting monolayers of BN, AlN, GaN, and InN are here systematically studied by *ab-initio* methods. We calculate the absorption spectra for in-plane and out-of-plane light polarization including quasiparticle and excitonic effects. Chemical trends with the cation of the absorption edge and the exciton binding are discussed in terms of the band structures. Exciton binding energies and localization radii are explained within the Rytova-Keldysh model for excitons in two dimensions. The strong excitonic effects are due to the interplay of low dimensionality, confinement effects, and reduced screening. We find exciton radiative lifetimes ranging from tenths of picoseconds (BN) to tenths of nanoseconds (InN) at room temperature, thus making 2D nitrides, especially InN, promising materials for light-emitting diodes and high-performance solar cells.

Quasiparticle Electronic Structure

The electronic band structures of the 2D group-III nitrides are displayed in Fig. SM1. Although their geometry resembles that of the graphene, the different electronegativities between nitrogen and B, Al, Ga and In atoms open significant gaps at the K points of the Brillouin zone (BZ). The conduction band minimum moves to the Γ point for all materials studied. In the case of the light cation, i.e., BN and AlN, the valence band maximum is fixed at K (as in graphene). However, in the case of the heavier cations, Ga and In, it also moves to the Γ point.

Direct allowed optical transitions are indicated in Fig. SM1 by vertical arrows for not too large interband energies. The lowest one occurs at Γ, independent of the cation. Higher-energy transitions are however possible at M and K between the uppermost valence band and the lowest conduction band. Transitions into the second-lowest conduction band may also occur at Γ, in particular for BN and AlN, where the energy distance to the conduction band minimum is relatively small compared to the gap size.

Optical Spectra

In order to characterize the linear optical properties of the monolayer nitrides, the real and imaginary parts of the 2D optical conductivity are presented in Figs. SM2 – SM5 for both in-plane and out-of-plane light polarization. The many-body effects are described in three different approximations:

(i) In the independent-particle approximation the electron-hole interactions are not taken into account. The single-particle eigenfunctions and eigenenergies are taken from the Kohn-Sham (KS) approach.

(ii) In the independent-quasiparticle (QP) approach the KS eigenvalues are corrected by QP shifts computed within the G_0W_0 approximation for exchange and correlation interactions. Correspondingly, the spectra are significantly blueshifted by about 2.5 eV (BN, AlN), 1.8 eV (GaN), and 1.0 eV (InN).

(iii) The BSE spectra including the screened electron-hole attraction and the unscreened electron-hole exchange interaction are characterized by three important features with respect to the independent-QP approach. There is, in general, a redshift accompanied by a spectral redistribution. The most striking feature is, however, the formation of bound exciton states below the QP gaps.

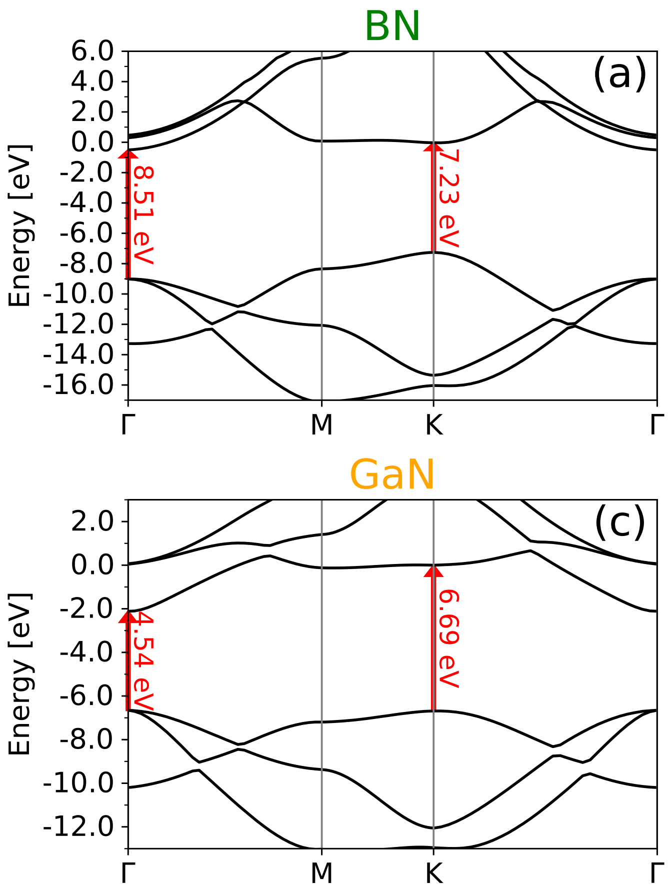

Figure SM1. Electronic quasiparticle band structures of (a) BN, (b) AlN, (c) GaN and (d) InN sheet crystals obtained within the one-shot G_0W_0 approximation. The vertical arrows indicate possible strong optical transitions in these 2D materials. The vacuum level position is used as energy zero.

These phenomena are visible in the real parts or the optical conductivity, especially by significant peaks, while in its imaginary parts still some resonance features are observable. The real parts vanish for small photon energies. Their slopes characterize the dielectric properties of the sheets.

The most important feature of changing the light polarization from an in-plane position to the (normal) out-of-plane direction is a significant blueshift of the spectra. It can be related to depolarization effects or local-field effects as described by the electron-hole exchange interaction in the pair Hamiltonian H_{exc}.

2D model Hamiltonian

For a better understanding of the lowest-energy ground state excitons with $S = 0$, $\mathbf{Q} = 0$ and excitation energy $E_0(0)$ with an approximate $1s$ character, we compare the *ab-initio* results for the exciton binding energy E_b and the exciton radius r_{exc} obtained within the accurate, but computationally heavy BSE calculations, with predictions of an analytical model of excitons in two dimensions[1,2]. Thereby, we focus on the bound excitons at the absorption edge. The Schrödinger equation, with excitonic eigenvalues E_n and eigenfunctions $\phi_n(\boldsymbol{\rho})$, can be formulated with an effective Hamiltonian as[2]

$$\left\{E_g - \frac{\hbar^2}{2\mu}\nabla^2_{\boldsymbol{\rho}} + W(\rho)\right\}\phi_n(\boldsymbol{\rho}) = E_n\phi_n(\boldsymbol{\rho}), \tag{SM1}$$

where the reduced exciton mass $\mu = m_e m_h/(m_e + m_h)$ is introduced within the effective mass approximation (EMA) with the electron (hole) mass m_e (m_h) derived from dispersion of the lowest conduction (highest valence) band. The relative distance

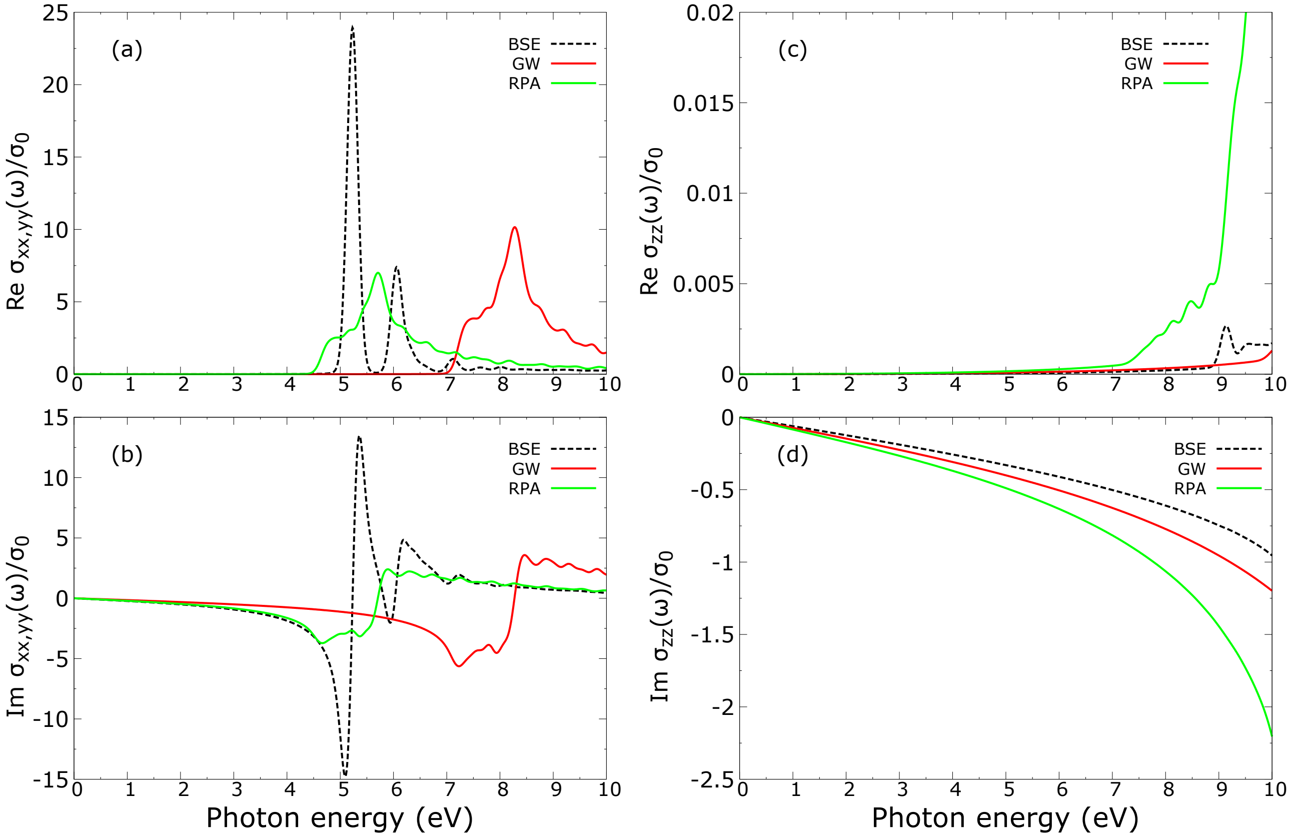

Figure SM2. Real (a,c) and imaginary (b,d) parts of optical conductivity for BN calculated within the independent-particle approach (RPA - green lines), within the independent-quasiparticle approach (GW - red lines) and taking into account excitonic effects (BSE - black dotted lines) for in-plane (a,b) and out-of-plane polarization (c,d).

between electron and hole is described by the vector $\boldsymbol{\rho}$. The screened Coulomb interaction is[2–4]

$$W(\rho) = -\frac{e^2}{4\alpha_{2D}}[H_0(\frac{\rho}{2\pi\alpha_{2D}}) - Y_0(\frac{\rho}{2\pi\alpha_{2D}})], \quad \text{(SM2)}$$

with H_0 and N_0 as the Struve and Neumann functions, respectively. $\alpha_{2D} = L \cdot (\mathrm{Re}\varepsilon_{\parallel}(\omega = 0) - 1)/4\pi$ denotes the 2D static electronic polarizability of the sheet, which is computed from the in-plane dielectric function $\varepsilon_{\parallel}$ in the limit of vanishing wave vector and frequency.

This exciton problem can be solved analytically just in the Mott-Wannier limit, i.e., for large electron-hole distances and/or vanishing screening. In fact, for $\rho/2\pi\alpha_{2D} \gg 1$, the unscreened 2D Coulomb potential

$$W(\rho) = -e^2/\rho. \quad \text{(SM3)}$$

appears, and the *e*-*h* pair feels a bare 2D Coulomb potential. In other words, in the Wannier-Mott limit a 2D exciton is described by an unscreened two-dimensional hydrogen-like system. The 2D hydrogen eigenvalue problem can be solved analytically[5], resulting in a huge binding energy $E_b = 4Ry\mu/m$ and a small *e*-*h* distance $r_{\mathrm{exc}} = a_B m/2\mu$ with the hydrogen Rydberg Ry and the Bohr radius a_B.

In the opposite limit of $\rho/2\pi\alpha_{2D} \ll 1$, i.e., in the limit of small electron-hole distances and/or large sheet polarizabilities, one finds a logarithmic behavior for the screened interaction, namely

$$W(\rho) = \frac{e^2}{2\pi\alpha_{2D}}\left[ln\left(\frac{4\pi\alpha_{2D}}{\rho}\right) - \gamma\right], \quad \text{(SM4)}$$

where $\gamma = 0.577$ is the Euler constant. The variational approach to this logarithmic limit gives as solutions $r_{exc}= a_{exc}/\sqrt{A}$ and $E_b=2R_{exc}A\ [\ln\frac{4}{\sqrt{A}} - \frac{3}{2}]$, with $A=a_{exc}/(2\pi\alpha_{2D})$ and $a_{exc}=a_B m/\mu$.

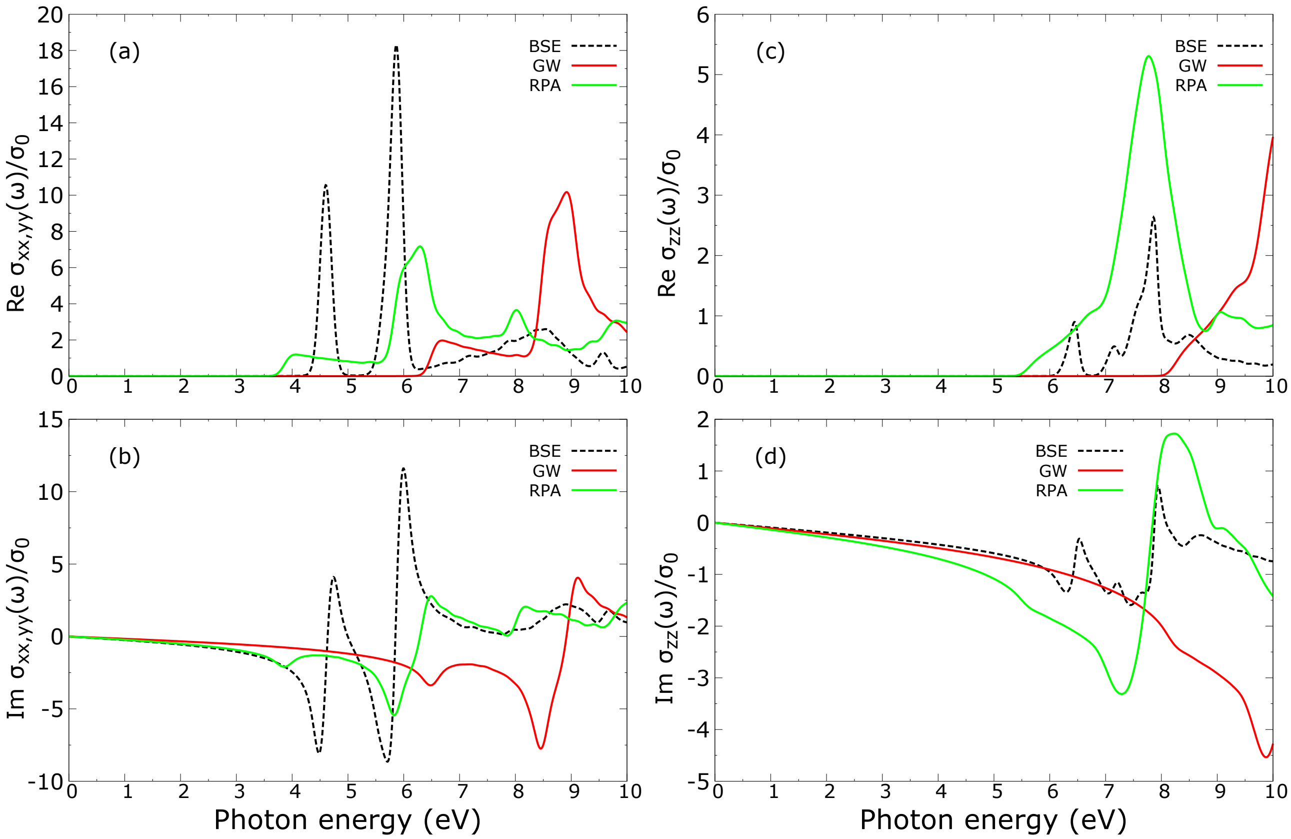

Figure SM3. Real (a,c) and imaginary (b,d) of optical conductivity for AlN calculated within the independent-particle approach (RPA - green lines), within the independent quasiparticle approach (GW - red lines) and taking into account excitonic effects (BSE - black dotted lines) for in-plane (a,b) and out-of-plane polarization (c,d).

We have solved the 2D model Hamiltonian within the variational approach, determining the excitonic radius and binding energy as reported in Table 1 of the manuscript. The graphical solution of the variational approach is shown in Fig. SM6. The black and red symbols indicate the variational solutions for each material. They all fall in an range intermediate between the Wannier-Mott (left side of the graphic) and the logarithmic one (right side of the graphic).

In order to be more quantitative, we compare the variational values of r_{exc} and E_b with those obtained within the logarithmic and within the Wannier-Mott limits. The results are shown in Table SM1. The values obtained for the excitonic radii and binding energies show that in our 2D III-N systems, the intermediate range is realized, i.e., neither the logarithmic, but especially nor the Wannier-Mott solution gives a good description of the BSE results (which represent our benchmark). In particular the Wannier-Mott results are very far from those obtained ab-initio within the BSE. The logarithmic results, instead, although showing sizeable differences, are much less dramatically far from the BSE values. The best agreement is found with the results obtained within the variational solution of the 2D model Hamiltonian with the potential (SM2).

	r_{exc}^{BSE} (Å)	E_b^{BSE} (eV)	r_{exc}^{ln} (Å)	E_b^{ln} (eV)	r_{exc}^{var} (Å)	E_b^{var} (eV)	r_{exc}^{2DH} (Å)	E_b^{2DH} (eV)
BN	3.8	2.0	3.1	1.4	3.7	2.1	0.7	20.8
AlN	4.5	1.9	2.9	1.6	3.4	2.3	0.6	23.6
GaN	8.0	1.2	5.3	0.7	6.6	1.4	1.5	9.4
InN	15.5	0.6	13.6	0.3	16.4	0.5	3.6	4.0

Table SM1. Excitonic radius r_{exc} and binding energy E_b for 2D III-nitrides. Columns 2, 3: results obtained within the ab-initio Bethe-Salpeter approach (BSE). Columns 4 and 5: results obtained by solving the 2D excitonic model hamiltonian in the logarithmic limit (ln). Columns 6 and 7: variational solution of the 2D model Hamiltonian. Columns 8 and 9: solution of the Wannier-Mott limit, that is the 2D unscreened hydrogenic model.

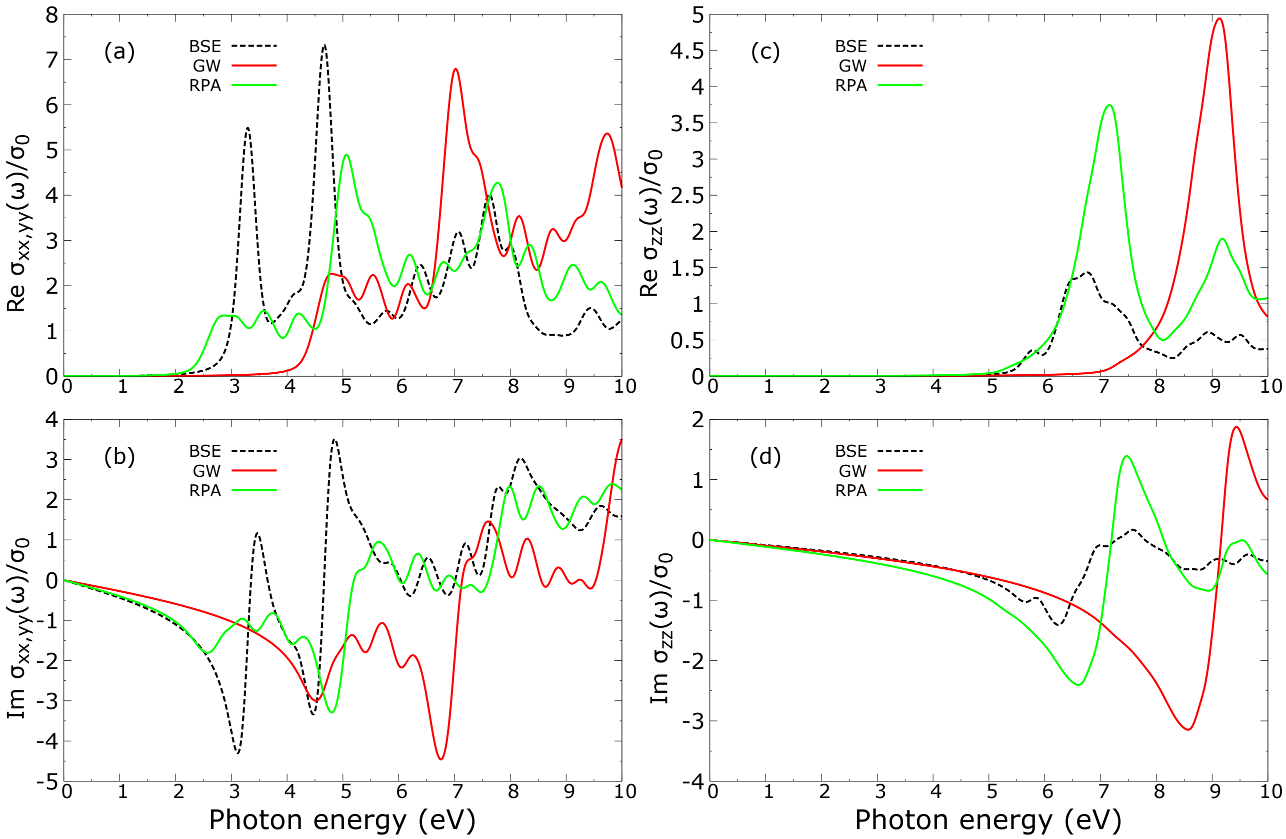

Figure SM4. Real (a,c) and imaginary (b,d) of optical conductivity for GaN calculated within the independent-particle approach (RPA - green lines), within the independent quasiparticle-approach (GW - red lines) and taking into account excitonic effects (BSE - black dotted lines) for in-plane (a,b) and out-of-plane polarization (c,d).

References

1. Cudazzo, P., Tokatly, I. V. & Rubio, A. Dielectric screening in two-dimensional insulators: Implications for excitonic and impurity states in graphane. *Phys. Rev. B* **84**, 085406 (2011).
2. Pulci, O. *et al.* Excitons in two-dimensional sheets with honeycomb symmetry. *Phys. Status Solidi (b)* **252**, 72–77 (2015).
3. Keldysh, L. Coulomb interaction in thin semiconductor and semimetal films. *JETP Lett.* **29**, 658 (1979).
4. Guseinov, R. Coulomb interaction and excitons in a superlattice. *Phys. Status Solidi (b)* **125**, 237–243 (1984).
5. Haug, H. & Koch, S. *Quantum Theory of the Optical and Electronic Properties of Semiconductors* (World Scientific, Singapore, 2009).

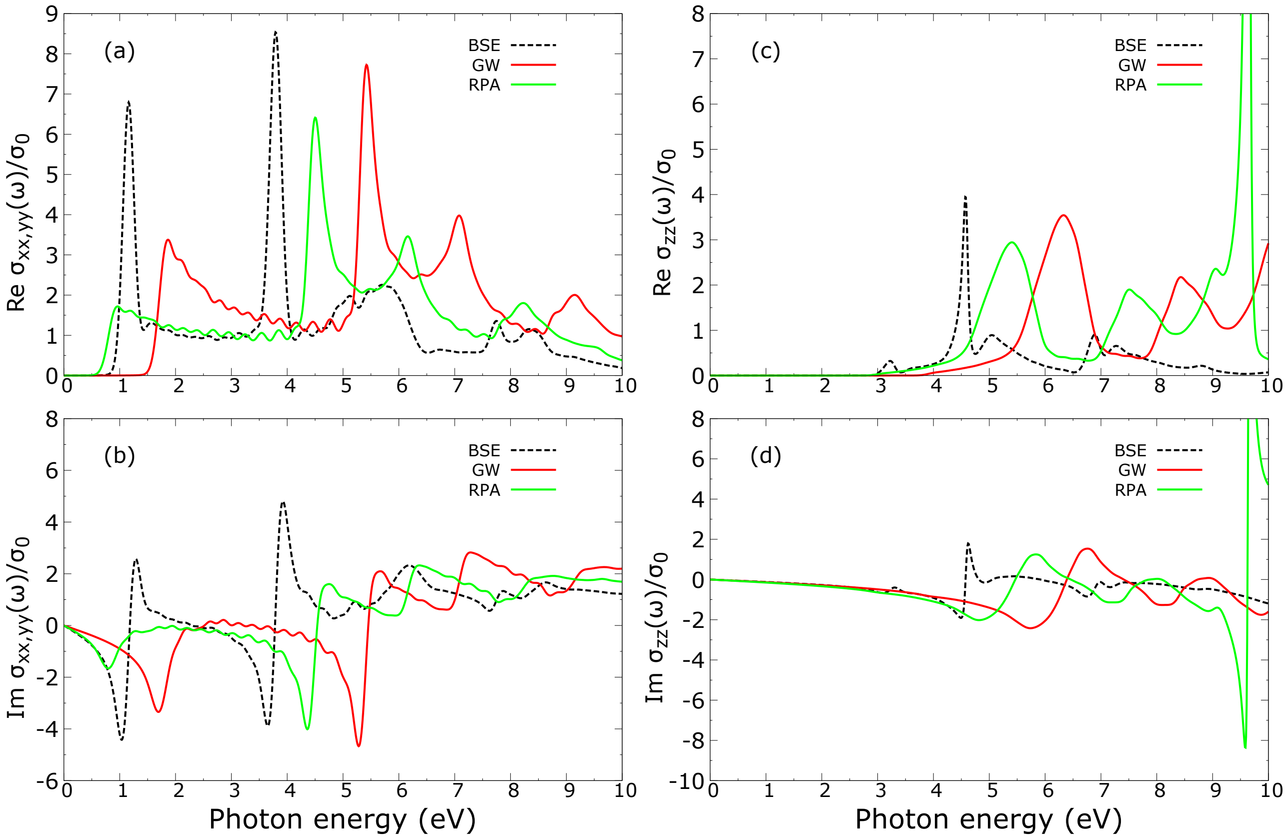

Figure SM5. Real (a,c) and imaginary (b,d) of optical conductivity for InN calculated within the independent-particle approach (RPA - green lines), within the independent quasiparticle-approach (GW - red lines) and taking into account excitonic effects (BSE - black dotted lines) for in-plane (a,b) and out-of-plane polarization (c,d).

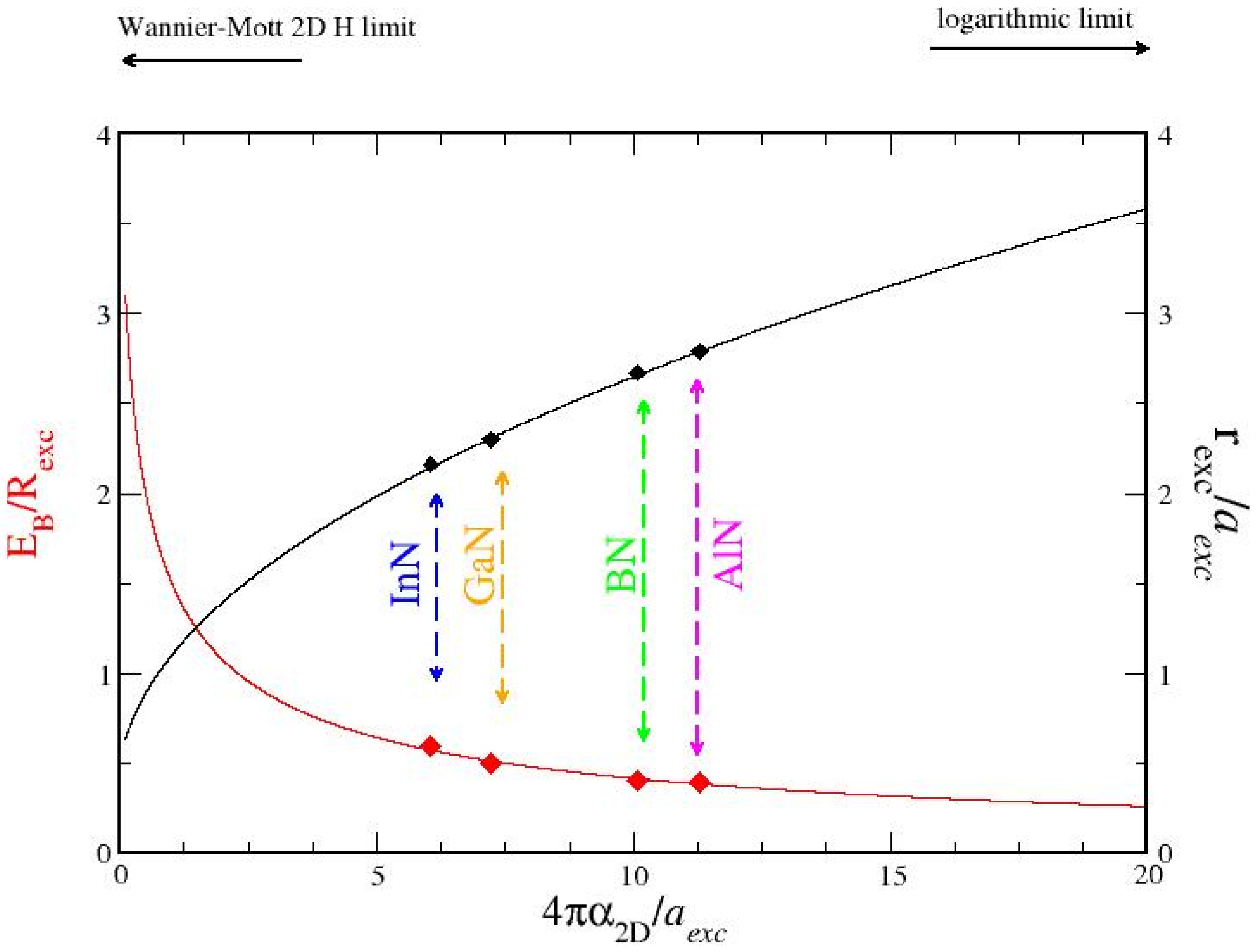

Figure SM6. Graphical variational solution of the 2D model Hamiltonian eq. (SM1) with the full potential eq. (SM2). The red curve gives the exciton binding energy E_b (renormalized to the excitonic Rydberg R_{exc}). The black curve gives the excitonic radius (renormalized to $a_{exc}=a_B m/\mu$). The left region of the graphic (small α_{2D}) describes the Wannier-Mott 2D hydrogenic limit of (eq. SM3), while the right region (large α_{2D}) the logarithmic limit (eq. SM4). Symbols indicate the results for the 2D Nitrides.
